# Performance of chromosomal microarray for patients with intellectual disabilities/developmental delay, autism, and multiple congenital anomalies in a Chinese cohort

**DOI:** 10.1186/1755-8166-7-34

**Published:** 2014-05-23

**Authors:** Wilson Wai Sing Chong, Ivan Fai Man Lo, Stephen Tak Sum Lam, Chi Chiu Wang, Ho Ming Luk, Tak Yeung Leung, Kwong Wai Choy

**Affiliations:** 1 Department of Obstetrics and Gynaecology, The Chinese University of Hong Kong, Hong Kong SAR, China; 2 Prenatal genetic diagnosis laboratory, Prince of Wales Hospital, 30-32 Ngan Shing Street, Shatin, Hong Kong SAR, China; 3 Clinical Genetic Service, Department of Health, Hong Kong SAR, China; 4 CUHK-Shenzhen Research Institute, The Chinese University of Hong Kong, Shenzhen, China; 5 Joint Centre with Utrecht University-Genetic Core, School of Biomedical Science, The Chinese University of Hong Kong, Hong Kong SAR, China

**Keywords:** Chromosomal microarray, Array CGH, Developmental delay, Intellectual disabilities, Multiple congenital anomalies

## Abstract

**Background:**

Chromosomal microarray (CMA) is currently the first-tier genetic test for patients with idiopathic neuropsychiatric diseases in many countries. Its improved diagnostic yield over karyotyping and other molecular testing facilitates the identification of the underlying causes of neuropsychiatric diseases. In this study, we applied oligonucleotide array comparative genomic hybridization as the molecular genetic test in a Chinese cohort of children with DD/ID, autism or MCA.

**Results:**

CMA identified 7 clinically significant microduplications and 17 microdeletions in 19.0% (20/105) patients, with size of aberrant regions ranging from 11 kb to 10.7 Mb. Fourteen of the pathogenic copy number variant (CNV) detected corresponded to well known microdeletion or microduplication syndromes. Four overlapped with critical regions of recently identified genomic syndromes. We also identified a rare de novo 2.3 Mb deletion at 8p21.3-21.2 as a pathogenic submicroscopic CNV. We also identified two novel CNVs, one at Xq28 and the other at 12q21.31-q21.33, in two patients (1.9%) with unclear clinical significance. Overall, the detection rate of CMA is comparable to figures previously reported for accurately detect submicroscopic chromosomal imbalances and pathogenic CNVs except mosaicism, balanced translocation and inversion.

**Conclusions:**

This study provided further evidence of an increased diagnostic yield of CMA and supported its use as a first line diagnostic tool for Chinese individuals with DD/ID, ASD, and MCA.

## Background

Array comparative genomic hybridization (aCGH) and single nucleotide polymorphism (SNP) genotyping array, collectively referred to as chromosomal microarray analysis (CMA), is commonly applied as a clinical diagnostic tool for patients with intellectual disabilities/developmental delay (ID/DD), autism spectrum disorders (ASD), and multiple congenital anomalies (MCA). With rapid advances in microarray resolution and throughput over the past few years, CMA has consistently shown a higher diagnostic yield than conventional karyotyping [1-5]. The International Collaboration for Clinical Genomics (ICCG), also known as International Standard for Cytogenomic Array (ISCA) Consortium, has recommended CMA over the karyotyping as the first-tier cytogenetic diagnostic test for patients with ID/DD and MCA [6]. Standards and guidelines recommended by American College of Medical Genetics (ACMG) have also been publised to standardise and improve the quality of CMA among different clinical genetic laboratories [7,8].

Rare copy number variants (CNV) have been implicated in the pathogenesis of many neuropsychiatric diseases despite the appreciation of the abundance of common CNVs in normal individuals [9,10]. Several studies have elucidated the causative role of CNV in DD/ID, ASD [11], congenital heart diseases [12], epilepsy [13], and congenital kidney malformation [14]. However, these studies also illustrated the phenotypic heterogeneity associated with a particular CNV. That is, the same CNV may confer risk to multiple diseases; other additional risk factors are required for the development of a specific disease outcome. This has led to the “two-hit” theory [15,16]. Under this circumstance, the clinical diagnosis, genetic counseling and management become challenging. An evidence-based approach which is dependent on the accumulation and delivery of knowledge through internet resources has been established to facilitate the result interpretations [17,18].

In this study, a high resolution 180 K oligonucleotide-CMA was applied in a Chinese cohort of patients with DD/ID, ASD, and MCA. The CMA findings were interpreted using the evidence-based workflow as recommended by ICCG and ACMG for molecular diagnosis of constitutional chromosomal and subchromosomal imbalances.

## Results

### CMA validation

To validate the CMA platform in this study, we first tested 10 cases of numerical chromosomal abnormalities including 7 trisomy cases (13, 16, 18, 21 and 22) and 3 monosomy cases (18, 21 and X). Subsequently 10 normal cases by karyotyping of normal individual were also included in the validation set. The CMA results were in 100% concordnace with karyotyping. The final set included 10 cases with known microscopic or submicroscopic chromosomal abnormalities (7 by karyotyping, 2 by multiplex ligation-dependent probe amplification, and one by multiplex ligation-dependent probe amplification and fluorescence in situ hybridization). Pathogenic CNV (pCNV) were detected in 7 cases (Additional file 1) and were concordant with the previous findings. The remaining cases without pCNV detected by CMA were mosaic marker chromosome, balanced structural rearrangement of chromosome 4, and balanced inversion of chromosome 12.

### Detection rate of pathogenic CNV in a postnatal cohort

A total of 105 patients with MR/DD/ASD/MCA referred to clinical genetics service were recruited for CMA application study. Custom-designed arrays precluding most of benign CNV as catalogued by Database of Genomic Variants (DGV) were applied in 67 patients (63.8%) while ISCA array were used in another 38 patients (36.2%). The average numbers of CNV found per patient using custom-designed array and ISCA array were 3 CNVs and 16 CNVs, respectively.

**Figure 1:**
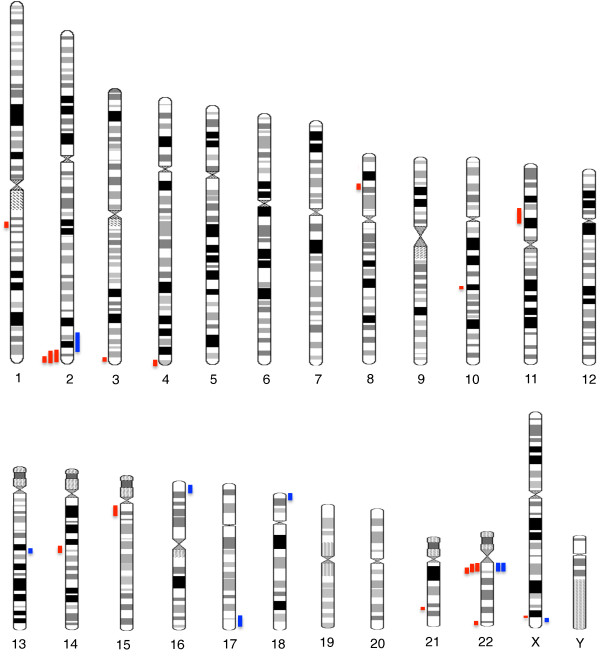
**An overview of pathogenic CNV found in this study.** Copy number loss is shown as red (left) and copy number gain in blue (right), respectively. The length of the bar indicating the size of each aberrant region.

The detection rates of pathogenic CNV from custom-designed and ISCA array were 17.9% and 21.1%, respectively, with an overall detection rate of 19.0%. A total of 24 CNVs (7 microduplications and 17 microdelections) in 20 patients were considered pathogenic (Figure 1). The size of the pathogenic CNV (pCNV) ranged from 11 kb to 7.1 Mb (Additional file 2). Recurrent pCNV were most frequently observed at the 22q11.2 region in this cohort, with two 22q11.2 duplications (MIM 608363) and three 22q11.2 deletions (Velocardiofacial/DiGeorge syndrome; MIM 188400) being identified. Deletions in the chromosomal region of 2q37.1-q37.3 known to cause the brachydacytly-mental retardation syndrome were the second most common pCNV. Three patients carried the submicroscopic deletions with an overlapping region of 2.7 Mb encompassing the critical *HDAC4* gene which is responsible for the phenotype. Three cases of pCNV each corresponding to Prader-Willi syndrome (MIM 176270), WAGR syndrome (MIM 612469), and 3q29 microdeletion (MIM 609425) were also identified.

Seven cases bearing rare CNV which overlapping with the critical regions of emerging genetic syndromes were identified and were considered pathogenic. These including five microdeletions (1q21.3-q22, 4q35.2, 10q23.1, 14q22.1-q22.3 and 21q22.13) and two microduplications (17q25.1-q25.3 and 13q21.2) as reported previously (Additional file 2). One patient was diagnosed with moderate DD, autistic features, hypotonia, and dermoid cyst over scalp. CMA showed a de novo 2.3 Mb deletion at 8p21.3-21.2 encompassing 37 genes. Although there was no known microdeletion syndrome associated with this region, we considered it pathogenic based on its size, de novo nature, and its overlapping with 70% of the pathogenic variant found in one DD + DM case (nssv578268) from the ISCA database and a similar 8p21 microdeletion being reported in a patient with ID and behavioral abnormalities.

There were two cases with duplication and deletion of size 1.33 and 6.22 Mb, respectively that were considered to bear a variant of unclear clinical significance (VOUS). The first case (aCGH3704) carried a 6.22 Mb deletion at 12q21.31-q21.33. The microdeletion was inherited from a physiologically healthy father and the patient does not carry any symptoms related to Bardet-Biedl syndrome. Bardet-Biedl syndrome is typically inherited in an autosomal recessive manner. For the second case (aCGH1293), it carries a 1.33 Mb microduplication containing the *MECP2* region. The X-chromosome inactivation (XCI) pattern has not been analyzed in the female patient and the *GDI1* gene was not involved, therefore we regarded these two microduplications as VOUS.

## Discussion

This is the first study describing the use of CMA in a Chinese cohort of patients with DD/ID, MCA, and autism referred for clinical genetics testing. The CMA platform was first validated in patients with known abnormal cytogenetic and molecular findings. Microscopic or submicroscopic chromosomal imbalances were accurately detected by CMA with improved resolution, the breakpoints of aberrant regions could be more precisely defined (Additional file 1). The advantage of increased precision in delineation of breakpoints facilitates the identification of the disease-causing genes [6]. The superiority of CMA over karyotyping has also been demonstrated in several cases. For instance, in a case of marker chromosome of unknown origin detected by karyotyping, CMA detected a 7.4 Mb duplication at 15q11.2-q13.1. This provided a clue to the origin of the marker chromosome and explained the phenotype of 15q11-q13 duplication syndrome as observed in this patient. In another patient with normal karyotype, CMA revealed a complex chromosome rearrangement involving a 10.7 Mb duplication at 2q36.1-q37.1 and a 9.7 Mb deletion at 2q37.1-q37.3. The size of individual aberration was clearly within the detection range by karyotyping (>5 Mb). However, the close proximity of these two aberrant regions resulting in small net gain of genomic materials (<1 Mb) which precluded its detection by karyotyping. In general, the limitations of CMA including its inability to detect low level mosaicism, heterochromatic abberrations, inversion and balanced translocation, were inherent to this platform [6,19].

In this study, twenty patients (19.0%) with DD/ID, ASD, and MCA were found to have at least one pathogenic CNV. The detection rate achieved in this study was similar to previous studies irrespective of the choice of oligonucleotide-based (13-28%) [20-26] or SNP-based platforms (15-19%) [27-29]. Improvement in the diagnostic yield of CMA is initially contributed by the increased genomic coverage and resolution of oligonucleotide arrays over bacterial artificial chromosome (BAC) arrays among aCGH platforms [30]. Both lower probe density and difference in genomic coverage may contribute to the lower detection rate. This is exemplified by the lower probe density custom designed 44 K array used in the majority of the cases which might contributed to a lower positive rate (17.9%) as expected compared to the higher density probe ISCA 180 K design (21.1%,). However, the smaller size of CNV identified were mostly benign. This is in concordance with previous reports indicated that most pathogenic CNV are >400 kb in size [6]. Our data suggested that appropriate size filter could be introduced to decrease the false positive calls.

Fourteen pCNV corresponded to the well known microdeletion or microduplication syndromes were identified. Velocardiofacial/DiGeorge syndrome, 22q11.2 duplication, brachydacytly-mental retardation syndrome, and Prader-Willi syndrome were among the most common recurrent genomic disorders observed in many different populations. They are known to be mediated by nonallelic homologous recombination (NAHR) between regions of segmental duplication. Our results also indicated that the pathogenic CNV associated with DD/ID, autism, and MCA were also largely contributed by NAHR in our study cohort.

We identified a rare microduplictaion (a *de novo* 2.3 Mb duplication) at 16p13.3 in a patient with dysmorphism, mild MR, camptodactyly, tracheomalacia in early childhood, blepharophimosis, and ptosis in adulthood. This region encompasses the *CREBBP* gene. Deletion of this gene is known to cause Rubinstein-Taybi syndrome [31] while its duplication is responsible for the observed phenotypes in chromosome 16p13.3 duplication syndrome which occurs in a frequency of 1 in 97,000 to 146, 000 live births [32].

Two CNV were classified as VOUS in two female patients. A de novo Xq28 duplication was identified in a patient with mild DD, DM, MCA, and hypotonia (Additional file 2). Xq28 duplication syndrome (MIM 300815) was first suspected given the cytoband location and the observed phenotype of DD. However, detailed genomic location as depicted by CMA showed that the critical 0.3 Mb region of Xq28 including the *GDI1* gene was not involved in our case. Instead, the duplicated region encompassed another set of genes which are known to cause several genomic syndromes only when they are deleted or mutated. In addition, there were no report of case with similar duplications from ISCA or DECIPHER database. In the second case, a paternally inherited deletion of 12q21.31-q21.33 was found in a patient with ID and DM. Although the father was phenotypically normal, the pathogenicity of this region could not be excluded. Homozygous deletion of *CEP290* (OMIM*610142) and *ALX1* (OMIM*601527) in this region are associated with different neurological diseases, but our case is a gain of copy number. Therefore, we considered these two microduplication regions as VOUS.

## Conclusions

In summary, our study has demonstrated the success of CMA application in patients with DD/ID, ASD, and MCA of a Chinese cohort. The interpretation of CMA findings was facilitated by publicly available databases, such as ISCA, DECIPHER, and DGV.

## Methods

### Patients

One hundred and fifteen patients with ID, DD, autism, or MCA from Clinical Genetic Service of the Department of Health of Hong Kong were recruited, both prospectively and retrospectively, in this study (IRB approval: LM/283/2010). All patients and their parents were studied by conventional karyotyping to exclude any inherited microscopic chromosomal abnormalities or balanced carrier status. Ten of them who had known genomic imbalances based on prior cytogenetic and/or molecular findings were chosen for platform validation. In addition, 10 prenatal samples with known numerical chromosomal abnormalities and 10 samples from adults with normal karyotype were recruited for the validation study. Another 105 patients without prior knowledge of genomic aberrations were recruited for application study and investigated by validated CMA. Informed consent was obtained from parents or guardians. Ethical approval of the study protocol was obtained from Ethics Committee of the Department of Health, Hong Kong. DNA was extracted from peripheral blood using DNeasy blood & tissue kit (Qiagen) according to manufacturer’s instructions. Genomic DNA concentration was measured by Nanodrop spectophotometer (ThermoFisher).

### CMA

The aCGH platform from Agilent Technologies was employed in this study. Custom-designed 44 K as described previously [33] and ISCA designed 180 K oligonucleotide microarrays were used. Both designs had similar genomic coverage for interrogation of over 100 genetic disorders but were different in probe density giving average genome-wide resolution of 100 kb and 25 kb, respectively.

Experimental procedures were performed according to manufacturer’s description. Briefly, one microgram of patient’s DNA and normal female control DNA (Promega) were differentially labeled with Cy5 and Cy3 respectively using Agilent SureTag Complete DNA Labeling Kit (Agilent Technologies, USA). Labeled DNA was then cleaned by purification columns (Agilent Technologies, USA) and hybridized on microarray for 24 hours. Microarray washing and scanning was performed using Agilent Oligo aCGH Wash Buffers (Agilent Technologies, USA) and Agilent Microarray Scanner (Agilent Technologies, USA) according to manufacturer’s instructions. All pathogenic CNVs were further validated on NimbleGen CGX-135 K array which were designed by Signature Genomics (Perkin Elmer, USA) following manufacturer’s instructions.

### Data analysis

Microarray images were processed with Feature Extraction v.11.1 (Agilent Technologies, USA) and imported to Agilent Genomic Workbench 7.0.4.0 for analysis. CNV were identified and curated to minimise false positive calls. Specifically, any aberration call with less than five probes and the value of log_2_ ratio in gain <0.25 or loss <0.5 was filtered. Parental aCGH were also performed to determine the origin of CNV if needed. CNV were considered pathogenic if they overlapped with the critical regions of well-characterized duplication/deletion syndromes or pathogenic regions as reported in ISCA or DatabasE of Chromosomal Imbalance and Phenotype in Humans using Ensembl Resources (DECIPHER) database, or were relatively large and encompassing many genes. Benign CNVs are those frequently seen among healthy individuals in the normal population as catalogued in the DGV. CNVs that contained a small number of genes but did not overlap with regions of known duplication/deletion syndrome, and that were not normal CNVs reported in the DGV, were regarded as VOUS (variant of uncertain clinical significance).

## Abbreviations

aCGH: Array comparative genomic hybridization; SNP: Single nucleotide polymorphism; CMA: Chromosomal microarray analysis; DD/ID: Developmental delay/intellectual disability; ASD: Autism spectrum disorders; MCA: Multiple congenital anomalies; ICCG: International collaboration for clinical genomics; ISCA: International standard for cytogenomic array; ACMG: American college of medical genetics; CNV: Copy number variant; pCNV: Pathogenic copy number variant; VOUS: Unclear clinical significance; BAC: Bacterial artificial chromosome; DM: Dysmorphism.

## Competing interests

The authors declare that they have no competing interests.

## Authors’ contributions

WC carried out the microarrays analysis and drafted the manuscript. IFML, HML, and STSL clinically examined the patients and collected clinical data. CCW performed molecular cytogenetic analysis and revised the manuscript. KWC and TYL conceived the work, and participated in its design, draft and revised the manuscript. All authors read and approved the final manuscript.

## Acknowledgements

This work was supported by Health and Health Services Research Fund (08090401) and the National Basic Research Program of China (81370715).

## Supplementary Material

Additional file 1 Validation of CMA platform using samples with abnormal results in previous test. Click here for file

Additional file 2 **CMA with pCNV or VOUS findings for chinese cohort of patients with intellectual disabilities/developmental delay, autism and MCA.** Notes: *NA* = not applicable [34-40]. Click here for file
